# Odor cues rather than personality affect tadpole deposition in a neotropical poison frog

**DOI:** 10.1093/cz/zoad042

**Published:** 2023-09-15

**Authors:** Mélissa Peignier, Max Ringler, Eva Ringler

**Affiliations:** a Division of Behavioural Ecology, Institute of Ecology and Evolution, University of Bern, CH-3032 Hinterkappelen, Switzerland; b Messerli Research Institute, University of Veterinary Medicine Vienna, 1210 Vienna, Austria; c Department of Behavioral and Cognitive Biology, University of Vienna, 1030 Vienna, Austria; d Department of Evolutionary Biology, University of Vienna, 1030 Vienna, Austria; e Institute of Electronic Music and Acoustics, University of Music and Performing Arts Graz, 8010 Graz, Austria

**Keywords:** animal personality, Anura, decision-making, parental care, tadpole transport

## Abstract

Animals constantly need to evaluate available external and internal information to make appropriate decisions. Identifying, assessing, and acting on relevant cues in contexts such as mate choice, intra-sexual competition, and parental care is particularly important for optimizing individual reproductive success. Several factors can influence decision-making, such as external environmental cues and the animal’s own internal state, yet, we have limited knowledge on how animals integrate available information. Here, we used an entire island population (57 males, 53 females, and 1,109 tadpoles) of the neotropical brilliant-thighed poison frog *Allobates femoralis* to investigate how 2 factors (olfactory cues and personality traits) influence the ability of males to find and use new resources for tadpole deposition. We experimentally manipulated the location of tadpole deposition sites and their associated olfactory cues, and repeatedly measured exploration and boldness in adult males. We further reconstructed tadpole deposition choices via inferred parent−offspring relationships of adult frogs and tadpoles deposited in our experimental pools using molecular parentage analysis. We found that the discovery and use of new rearing sites were heavily influenced by olfactory cues; however, we did not find an effect of the measured behavioral traits on resource discovery and use. We conclude that in highly dynamic environments such as tropical rainforests, reliable external cues likely take priority over personality traits, helping individuals to discover and make use of reproductive resources.

On a daily basis, animals need to carefully balance risks and costs of their behavioral actions to maximize fitness (McFarland 1977). Several factors play a role in individual decision-making, such as not only the external cues (e.g., from the natural and social environment) but also the internal state of the individual (e.g., physiology, motivation) (McFarland 1977; Budaev et al. 2019). Consistent between-individual differences in behavior, also called “animal personality,” have been shown to explain between-individual differences in decision-making (Carter et al. 2013). Boldness, for example, has been shown to influence the likelihood of migration (Chapman et al. 2011) or foraging efficiency (Mella et al. 2015), while activity can influence the amount of risk taken when foraging (Boon et al. 2008). However, so far we have a limited understanding of how cue assessment and use differ between individuals with different personalities.

Decision-making is particularly relevant in the contexts of mate choice, intra-sexual competition, and parental care, due to the expected high impact on fitness (Clutton-Brock 1991). Parental care is defined as any parental behavior that increases offspring survival, while parents often bear some costs (i.e., reduction in survival or future reproductive success; Smiseth 2019). Therefore, appropriate decision-making is essential to optimize the costs and benefits of care to ensure offspring survival. Behaviors related to parental care can range from protection against predators to the exploitation of certain resources for the benefit of the offspring (e.g., foraging, discovery and use of rearing sites, and shelters; Royle et al. 2012). Individuals may vary in their execution of and performance in these behaviours, for example, in how successful certain individuals are in discovering new resources. Previous studies have shown that personality traits can impact decision-making in the context of parental care. For example, more aggressive male blue tits *Cyanistes caeruleus* feed their offspring at lower rates compared to less aggressive males (Mutzel et al. 2013). Bold chestnut thrush *Turdus rubrocanus* females choose nest sites with lower nest density, which, in turn, results in an increase in the number of nestlings but a decrease in nestling mass (Zhao et al. 2016).

The influence of decision-making in the context of parental care is even less studied in amphibians despite the great variety of parental care they show. Many neotropical poison frog species (Dendrobatidae, AmphibiaWeb 2023) lay their eggs on land and transport the newly hatched tadpoles to patchy water resources (Lötters et al. 2007; Stynoski et al. 2015). The ability of adults to find suitable water bodies is vital for their fitness, but tadpole transport is expensive in terms of time and energy invested, and probably risky (e.g., predation, loss of resources) for the transporting parent (Pough and Taigen 1990; Wells 2007; Pašukonis et al. 2019, 2022; Carvajal-Castro et al. 2021). Furthermore, tropical rainforests are highly dynamic environments and the availability and quality of suitable rearing sites might change unpredictably (Rudolf and Rödel 2005; Schulte and Lötters 2013; Fouilloux et al. 2021). Therefore, individuals should rely on all available information to find, assess, and choose the appropriate rearing sites that maximize offspring survival (McKeon and Summers 2013; Poelman et al. 2013; Ringler et al. 2013b, 2018). Previous studies have highlighted the importance of olfactory cues (external cues, e.g., Schulte et al. 2011; Rojas 2014; Schulte and Lötters 2014; Serrano-Rojas and Pašukonis 2021), natal site imprinting, and spatial memory (internal states, e.g., Ringler et al. 2013b, 2018; Erich et al. 2015; Pašukonis et al. 2016, 2019; Beck et al. 2017) for tadpole transport in poison frogs. How personality affects pool discovery and use has previously not been explored.

Here, we used a free-ranging population of the neotropical brilliant-thighed poison frog (*Allobates femoralis*, Dendrobatidae, AmphibiaWeb 2023) to investigate how external cues and personality traits influence the ability of individual males to find and use new rearing sites. We experimentally manipulated the position of tadpole deposition sites and the olfactory cues they emitted. We repeatedly measured exploration and boldness in adult, territorial males. We also collected tissue samples of adult frogs and tadpoles, and reconstructed tadpole deposition choices via inferred parent− offspring relationships of adult frogs and tadpoles deposited in our experimental pools using molecular parentage analysis. We expected more explorative and bolder males to find new rearing sites more quickly, use higher numbers of different rearing sites, and/or discover rearing sites located farther away from their home territory compared to less explorative or shyer males. Previous studies have hinted toward the importance of olfactory cues associated with stagnant water and/or tadpole odor for the initial discovery of new rearing sites (Pašukonis et al. 2016; Serrano-Rojas and Pašukonis 2021). Hence, we also expected pools with odor cues to be detected earlier than pools without any odor cues. We further investigated if certain personality types (e.g., less explorative individuals) are more likely than others to make use of odor cues during tadpole transport.

## Materials and Methods

### Study system

*Allobates femoralis* is a diurnal neotropical poison frog with a highly promiscuous mating system (Montanarin et al. 2011; Ursprung et al. 2011a; Stückler et al. 2019). During the reproductive season, males are highly territorial and perform advertisement calls from elevated perches to repel male competitors and attract female mating partners (Hödl et al. 2004; Ringler et al. 2011). Females commute from their perching site to males within a 20 m radius to mate and lay clutches in the leaf litter inside male territories (Ringler et al. 2012; Fischer et al. 2020). Females can produce a clutch every 8 days while males are known to care for up to 5 clutches at the same time (Weygoldt 1980; Ursprung et al. 2011a). The reproductive season lasts throughout the entire rainy season (early December until July in French Guiana) but typically reproduction decreases during periods with less rain (in French Guiana a 2−3 weeks short dry period in March). Thus the total number of clutches per individual is likely strongly influenced by the length of the rainy season in any given year. After 15−21 days of larval development, males provide care by transporting the newly hatched tadpoles to water bodies located up to 200 m outside of their territory where tadpoles will complete their development to metamorphosis within 40−50 days (Ringler et al. 2013b, 2018). Males actively spread larvae from single and successive clutches across several water bodies (Erich et al. 2015). In our study population on a small river island, the pools occupied by *A. femoralis* larvae are generally not used by other species, with the exception of the para toad *Rhinella castaneotica*, but resources (e.g., food) do not seem to be a limiting factor for both detrivorous species. Tadpoles of the dying poison frog *Dendrobates tinctorius*, are known to feed on *A. femoralis* larvae (Fouilloux et al. 2021), however, this and other species with carnivorous tadpoles are absent from the study island, while *A. femoralis* itself is not cannibalistic. Previous studies have shown that some males prefer returning to their natal pools despite the presence of predators, and even when closer pools are available (Ringler et al. 2018). Males display personality along the aggressive/docile, bold/shy, and exploratory/stationary axes (Chaloupka et al. 2022; Peignier et al. 2022).

### Frog population survey

We conducted our study using a wild population of *A. femoralis* located on a ~5 ha river island in a lowland rainforest, in the vicinity of the field camp “Saut Pararé” of the “CNRS Nouragues Ecological Research Station” in the nature reserve “Les Nouragues” in French Guiana; 4°02′N, 52°41′W (Bongers et al. 2001; Ringler et al. 2016). The population had been introduced in 2012 and it has been stable since then at ~150 adult individuals (Ringler et al. 2015). We surveyed the frog population on the island every day from 0900 to 1800 h between February and April 2019, and caught all encountered adults, aiming for total sampling of adult males and females on the island in this period. We collected tissue samples (i.e., toe clip) of all newly encountered adults for genetic analysis by removing the third toe of both hind limbs which we immediately preserved in 96% ethanol (Ursprung et al. 2011b). We also identified all adult frogs via digital pictures of their distinct ventral patterns and with the help of the pattern-matching software Wild-ID (Bolger et al. 2012). We sexed them by the presence (males) or absence (females) of vocal sacs. We recorded the exact spatial locations of all frogs on a digital map using rugged Win10 tablets (CAT T20, Bullitt Group, Reading, United Kingdom) with the mobile GIS software ArcPad 10 (ESRI, Redlands, CA, United States), and further handled the data in ArcGIS 10.6 (ESRI).

### Rearing pools set-up

**Figure 1:**
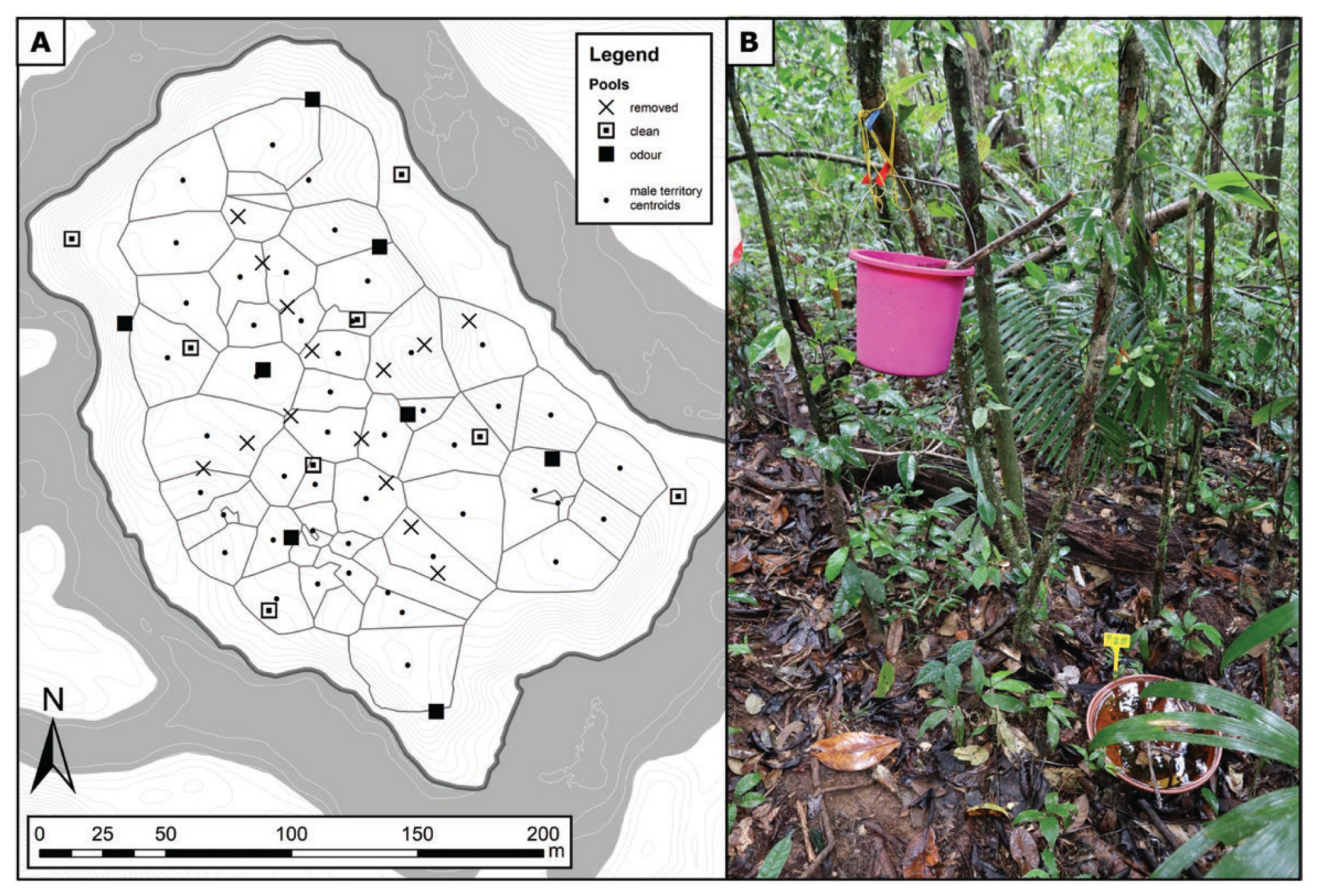
Map (A) showing the spatial distribution of male territories (delimited by black lines) and pool locations, and picture of the pool setup (B). On the map, the crosses represent the old pools that were removed at the beginning of the field season, while the squares represent the new pools with (black squares) or without (white and black squares) odor cues. The centroids of male territories are represented by a black dot. The map uses the Voronoi territories of the day when the most individuals were present at the same time on the island (7 March 2019). The thin gray lines represent the 50 cm elevation isoclines, and the dark gray area represents the river Arataye.

Until our field season in 2019, a cross-shaped array of 14 artificial pools (plastic bowls volume: ~15 L) that the frogs used for tadpole deposition were placed across the island. These artificial pools are necessary for the species to survive on the island that features very few natural sites that could serve for tadpole deposition otherwise. At the beginning of February 2019, we sampled all tadpoles from these pools, which we then removed (hereafter referred to as “old pools”). We then positioned 16 new pools (plastic pot trays, volume: ~5 L, with a similar surface but shallower than the old pools) in a cross-shaped array, turned by 45° in relation to the old pools (Figure 1A; hereafter referred to as “new pools”). We filled all new pools with rainwater and added a few leaves from the surrounding forest floor. At the end of the study period, we put the 14 old pools back at their original location.

To identify factors influencing tadpole deposition and pool discovery, we collected tissue samples from all tadpoles by clipping the tail tips of all individuals and preserving them in 96% ethanol. We sampled tadpoles from the new pools every day from 15 February to 2 April 2019, and from 6 to 22 April 2019. We also did one additional sampling in May 2019 to obtain information about the temporal sequence of successful deposition events. To be able to identify the sequential order of tadpole depositions on a daily basis, we did not return tadpoles to their original pool after sampling. We distributed these tadpoles evenly across 5 L buckets that were suspended 1 m above the ground and filled with 5 L of rainwater and 2 L of leaves from the litter underneath so that all buckets received the same number of tadpoles (Figure 1B). This way, no transporting adult male could deposit new tadpoles inside these buckets, but tadpoles were able to complete their development until hatching.

The buckets were positioned within 1 m of every other new pool to prevent tadpoles from accidentally dropping into new pools and to create an odor plume associated with half of the new pools (Figure 1). This setup was intended to assess long-range pool finding/use by creating odor plumes acting as long-distance attractants to transporting frogs and resembled the one from Pašukonis et al. (2016) where an attractive effect of such a suspended tadpoles-filled bucket was found. The new pools with the nearby overhanging buckets served as treatment sites “with odour cues,” while the other pools were taken as controls. Control pools did not have any overhanging buckets, as we deemed the possibility that accumulating water and organic matter would have provided similar odor cues than the treatment sites more relevant than the possibility that such buckets at 1 m height could provide relevant visual cues for a strictly terrestrial species. We also exchanged the water in new pools with rain water on a regular basis to prevent desiccation, colonization of the pools by predatory dragonfly larvae and the accumulation of odor cues from tadpoles and leaves (cf. Serrano-Rojas and Pašukonis 2021 for the attractive effect of stagnant water and fermented leaves).

### Parentage analysis

We identified individual deposition patterns via pedigree reconstruction, using molecular parentage analysis. We also had access to genetic data of older individuals (survivors from previous years, found again in 2019) from the long-term monitoring on the island population. Our sample comprised a total of 121 adults (64 males and 57 females) and 1,142 tadpoles.

We isolated genomic DNA of all samples using a Proteinase K digestion followed by an extraction using a DNeasy kit (QIAGEN, Valencia, CA, USA). We then amplified all samples at12 highly variable microsatellite loci (Afem03, Afem04, Afem05, Afem09, Afem12, Afem13, Afem16, Afem20, Afem22, Afem24, Afem25, Afem27) using fluorescent-labeled primers and PCR protocols described in Jehle et al. (2008) and Ringler et al. (2013a). Finally, we diluted the amplified products with water, mixed them with internal size standard LIZ, and ran them on a capillary sequencer (ABI 3730, Applied Biosystems/Thermo Fisher Scientific, Waltham, MA, USA). We visually identified all loci and determined the allele sizes using PeakScanner 1.0 (Applied Biosystems). We determined the final allele sizes using the binning software Tandem 1.01 (Matschiner and Salzburger 2009). We removed individuals for which 4 or more loci could not be scored. In total, we performed the pedigree reconstruction on 57 males, 53 females, and 1109 tadpoles.

We conducted parentage assignments using the software COLONY 2.0.6.7 (Jones and Wang 2010). We built a full likelihood model allowing for polygamous mating in both sexes with a medium precision and without setting a sibship prior. To determine deposition patterns, we treated individual adults as potential “fathers” and “mothers,” whereas all tadpoles were treated as potential “offspring.” We used the “Best (ML) Configuration” for further analyses. Of the 1,109 tadpoles, COLONY assigned 1,006 individuals (90.7%) to at least one known parent. For 695 of these 1,006 individuals, both parents were assigned (69.1%). When one or both parents were not found within our sampled adult genotype dataset, the parental genotype was simulated by the software. For all subsequent analyses, we only used tadpoles for which at least the father was known (*N* = 898 tadpoles from 52 fathers). Since *A. femoralis* female can lay a clutch every 8 days on average (Weygoldt 1980; Fischer et al. 2020), we assumed that tadpoles from the same parent pair that were deposited in one or several pools within 6 days belonged to the same clutch. We also considered that tadpoles deposited on a specific day with only the father assigned belonged to the same clutch as tadpoles from the same day where both father and mother were assigned, as it is unlikely that a male will transport 2 different clutches in a single day (Ringler et al. 2013a). Instead, it is more likely that we were not able to score enough alleles to properly identify the female for all tadpoles. We made this conservative choice to avoid overestimating the number of clutches produced by each male. This case happened 15 times out of 125 clutches. Finally, we considered that tadpoles produced by the same parent pair that were deposited more than 6 days apart were from 2 separate clutches.

Males typically distribute their clutches across several pools, possibly to improve offspring survival (Erich et al. 2015). Therefore, to quantify parental performances, we determined how many different old and new pools each individual used, pre- and during-treatment, respectively. We also identified which males were the first to discover a new pool by determining if males deposited a clutch in a new pool within 45 days of installation (1) or not (0). We chose this timeframe to represent half of our study period, thus giving enough time for some individuals to discover and use the new pools. While this measure depends on whether a male had obtained one or several clutches to deposit within the first 45 days or not, we do not expect this to considerably bias our results. Our study was performed at the peak of the rearing season, and almost all males had already produced clutches at that time; thus, we considered that sufficient males had clutches to end up in our sample for a representative coverage of reproducing males.

### Spatial analysis

To understand which factors affect pool discovery and choice of transporting males, we determined for each clutch in which pool(s) it was deposited, if the pool site featured odor cues or not, the distance between the pool and the male’s territory (centroids), and the distance between the new pool and the closest old pool that had been removed (as a proxy for potential memory of old pools). These parameters were used to verify if males discovered new pools by searching close to their territory or close to old pools (cf. Pašukonis et al. 2016 for searching behavior at former pool sites). Additionally, we measured the average distance between the male territories (centroids) and each one of the old pools to verify if the disturbance we created by removing rearing sites in the peak of the mating season changed the dynamic of tadpole deposition (e.g., if there was a link between personality traits and paternal performance before but not after pool removal).

To determine daily territory centroids of males, we used the daily male territories that had been estimated in a previous study for the same time period (for details see Peignier et al. 2022). In this previous study, Dirichlet tessellation was used to approximate male territories as Voronoi polygons (Voronoi 1908) on a day-to-day basis. All capture points of the last 5 days a male was seen, including the focal day, but excluding all points where a male was likely found outside its territory were used (i.e., all capture points linked to tadpole transport). The vertices of the island outline were also included to avoid overestimating peripheral territories as *A. femoralis* tend to avoid the forest edges (Ringler et al. 2009). After conducting the Dirichlet tessellation, the resulting Voronoi polygons were dissolved based on male identity to obtain “Voronoi territories” for each male. We then used the corresponding function in ArcGIS 10.6 (ESRI) to determine daily territory centroids.

### Assessment of the level of exploration and boldness

For this study, we used the data and their analyses from a previous study on levels of exploration and boldness in the same *A. femoralis* population (Peignier et al. 2022). This previous study had been conducted concurrently to the pool discovery and choice experiment presented in this study. Its aim was to investigate the prevalence of personality (i.e., the repeatability of the behaviors and their structure into functional units). To this end, a novel environment test (NET), consisting of a cooler box with a 10 cm PVC tube attached on one side of the box (for details see Peignier et al. 2022), was used. Individuals were captured inside their territory and tested immediately on site. They were placed in the PVC tube for 10 min before a door separating the tube and the box was opened. Their behavior was filmed using a GoPro camera attached to the lid of the box for the next 15 min. Individuals were free to remain inside the tube, go into the box, or return to the tube at any time. Males were tested repeatedly, and it was aimed to test all individuals 3 times. The minimum duration between 2 consecutive tests with the same individual was 24 h (on average tests were 11.36 ± 8.43 standard deviation [*SD*] days apart). In total, 156 NET trials with 50 males (mean ± *SD* = 3.31 ± 1.50 repetitions per individual) were conducted. Individuals were always released immediately after the test and resumed normal behavior (e.g., calling activity) shortly after.

The likelihood and latency to emerge from the tube and the number of jumps performed in the novel environment were measured using the coding software BORIS (Friard et al. 2016). Furthermore, the distance traveled (in pixels) and the area covered in the novel environment were measured using the automated tracking software TOXTRAC (Rodriguez et al. 2018; Peignier et al. 2022). Individuals who did not emerge from the shelter were given a censored value of 900 s (total duration of the experiment).

Results from this previous study showed that all behaviors measured were repeatable. Furthermore, by applying structural equation modeling on the phenotypic covariance matrix derived from the means of each behavior for each individual, Peignier et al. (2022) showed that the best structural equation model supported the existence of 2 latent variables. The first latent variable explained the covariance between boldness-related behaviors (e.g., the likelihood and the latency to exit the tube). The second latent variable explained the covariance between exploration-related behaviors (e.g., the distance traveled, the area covered and the number of jumps performed in the novel environment). The two latent variables were correlated (Figure 3B; in Peignier et al. 2022). Based on these results, in the present study, we decided to use factor scores extracted from the best structural equation model as proxies for the exploration and boldness score of each individual.

### Statistical analysis

We conducted all statistical analyses in R v3.6.0 (R Core Team 2022) using the integrated development environment RStudio v1.3.1093 (RStudio Team 2019).

#### Influence of personality traits on paternal care

We used a bivariate approach to study how behaviors measured in the NET correlate with variation in paternal performances at the between-individuals level using Bayesian Generalized Linear Mixed Models (package “MCMCglmm,” Hadfield 2010). We constructed 5 bivariate models to quantify the relationship between exploration (factor score extracted from the best structural equation model in Peignier et al. 2022) and 1) the number of old and 2) new pools used, 3) the likelihood to discover a new pool within 45 days of installation, and 4) the average distance between the male’s territory and the old, and 5) new pool he deposited in. We also constructed an additional 5 bivariate models to look at the relationship between boldness (factor score extracted from the best structural equation model in Peignier et al. 2022) and the 5 paternal performance variables listed above.

In order to investigate between-individual covariance between exploration/boldness and the paternal performance variables, we divided each of the paternal performance variables by their mean value before adding them as the second response variable (see Houslay and Wilson 2017). We used the function “transformTukey” to apply a constant transformation on the distance and the time spent in the shelter to better approach a normal distribution. Models with the likelihood of discovering a new pool within 45 days were fitted as binomial. We further used the posterior distributions to estimate the between-individual correlations and covariances between personality and paternal performance.

We ran the models using a parameter expanded prior (described in the supplementary material), with 1,000,000 iterations, a burn-in of 10,000, and selected every 500th posterior parameter sample (thinning interval). We confirmed the absence of autocorrelation (correlation between lags <0.1), sufficient mixing (visual inspection of plots of MCMC chains), and that we ran the Markov chain for long enough (Heidelberg and Welch diagnostic tests; Hadfield 2010). We present estimates and credible intervals generated from our models, and we estimated that statistical significance was reached if the 95% credible intervals did not overlap 0.

#### Influence of external cues on paternal care

Next, we identified the parameters that best predict patterns of tadpole deposition in a given pool. To this end, we structured the data in a way that included all possible deposition options (i.e., all available 16 new artificial pools) of a given tadpole transport event (i.e., the deposition of tadpoles from one single clutch across single or multiple pools) and noted whether the assigned father deposited tadpoles at a given pool or not (“deposition”: yes = 1/no = 0). We further included the “distance between the pool and the male’s territory,” the “distance between the pool and the previously closest (used or not) old pool,” the male’s and clutch identity, the male’s exploration and boldness scores, and the treatment of the pool (with or without odor cues) in the data set.

We fitted a generalized linear mixed effect model (GLMM) using the package *lme4* (Bates et al. 2016), with “deposition” as the response variable. As fixed effects, we included “treatment” (pool with or without odor cues), “distance between the pool and the male’s territory,” and “distance between the pool and the closest old pool.” The parameters “clutchID” nested within “maleID” were used as random factors. We conducted a likelihood ratio test based on the maximum likelihood fits of the full and the null model using the “fixedLRT” function to obtain *P* value estimates for the overall fit of the model. Additionally, we performed a Conditional Inference Tree using the package “partykit” to better understand the ranking based on relative importance of the fixed effects (Hothorn and Zeileis 2015). We used the same response and partitioning variables as for the GLMM (fixed effects) to grow the conditional inference tree (Hothorn et al. 2006).

#### Influence of the interplay between personality traits and external cues on paternal care

We looked at the interplay between olfactory cues and personality traits in paternal care. We fitted two GLMMs with a Poisson distribution using the number of new pools as the response variable, and two further GLMMs with a binomial distribution using the likelihood to discover a new pool within 45 days as the response variable. For each model, we included 2 two-way interactions as fixed effects and male identity as the random effect. One interaction was between the factor score of exploration and either “treatment” or the scaled “distance between the pool and the male’s territory.” The other interaction was between the factor score of boldness and either “treatment” or the scaled “distance between the pool and the male’s territory.” The 4 models were tested for multicollinearity using the function “vif,” with scores above 5 indicating that two or more predictor variables were correlated to each other (James et al. 2014). Some multicollinearity was detected in both models with “odour cues” as a fixed effect (see Supplementary Table 1). To confirm our results, we run an additional analysis and split these models in two, with either only the 2-way interaction between exploration and odor cues or only the 2-way interaction between boldness and odor cues.

## Results

**Figure 2:**
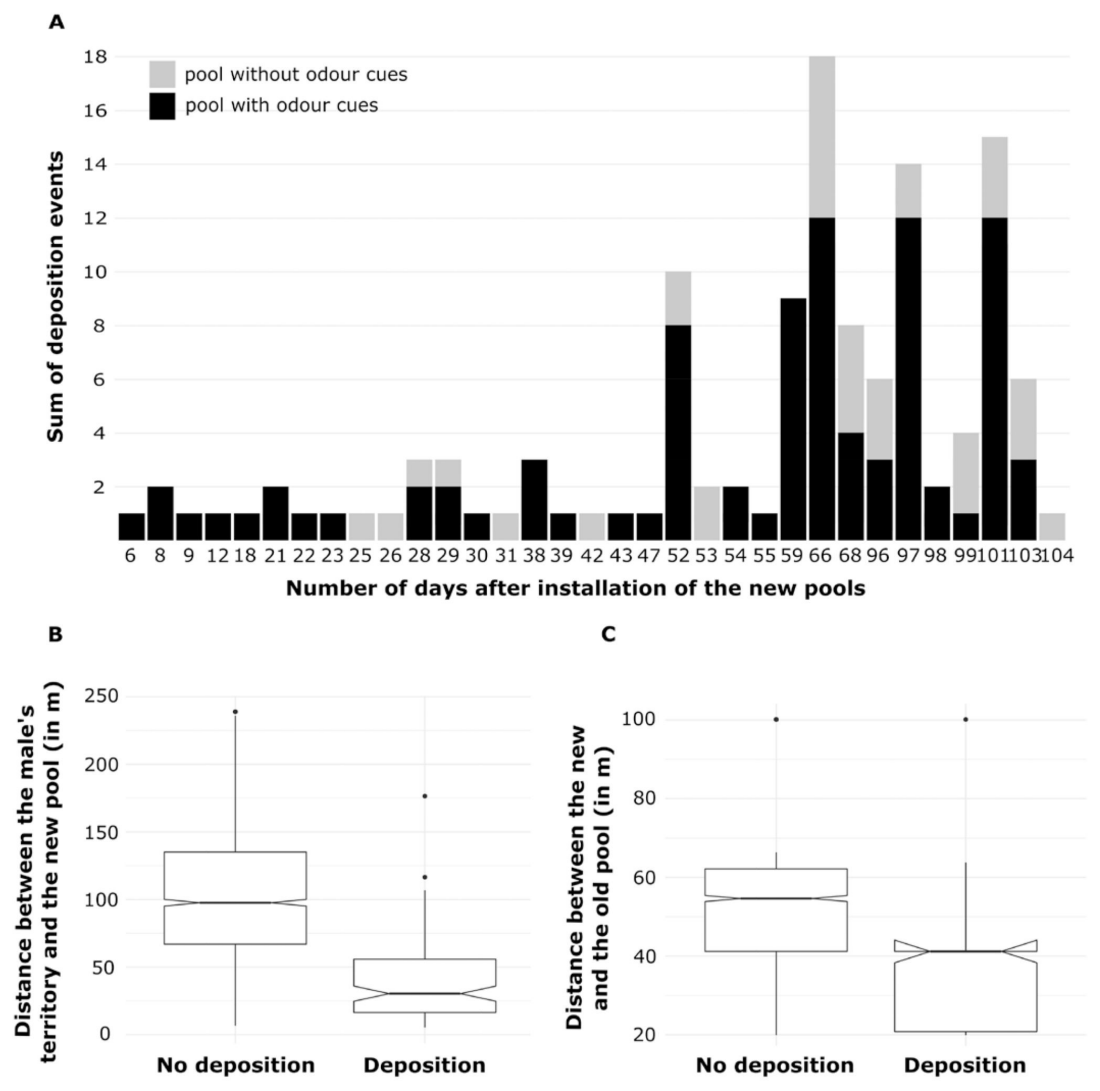
Patterns of discovery and use of new rearing resources for tadpole deposition. (A) Temporal sequence of deposition events in pools with (black) and without (light gray) odor cues. Pools were sampled daily from the 1st to the 47th day after installation, then from the 51st to the 68th day and from the 95th to the 103rd day post-installation. (B) Boxplots displaying pools in which males choose to deposit or not based on their distance to the centroid of the male’s territory. (C) Boxplots showing pools in which male choose to deposit or not based on their distance to the closest old pool. In (B) and (C), the median is represented by the middle horizontal line, the interquartile range is represented by the upper and lower edges of the boxplots, the qualitative difference in median is represented by the notches, and the upper and lower quantiles. (1.5 × inter-quartile range) are represented by the whiskers.

Of the 57 males and 53 females sampled, 52 males (91.2%) and 47 females (88.7%) produced at least one tadpole in 2019. We found deposited tadpoles of 37 males in the old and 41 males in the new pools. We collected behavioral data on 45 out of the 52 individuals but did not manage to test the 7 other males. Of the 16 new pools installed 12 pools were used for tadpole deposition at least once. All depositions that occurred within the first 3 weeks after the opening of the new pool sites happened in pools with odor cues (Figure 2A). Only after 3 weeks did males start to discover and use the control pools. Only 10 males out of 41 discovered and used new pools within the first 45 days of installation. On average, males moved 98 m to deposit their tadpoles (±49 m *SD*), and used pools were located between 19 and 100 m (average = 48 m, *SD* = 21 m) from an old pool.

### Influence of personality traits on paternal care

There was no correlation between levels of exploration or boldness and pool use at the between-individuals level (Supplementary Table 2). Males with varying levels of exploration or boldness did not differ in the number of pools they used, how far from their territory they went to deposit the tadpoles, or how quickly they discovered the new pools.

### Influence of external cues on paternal care

**Figure 3:**
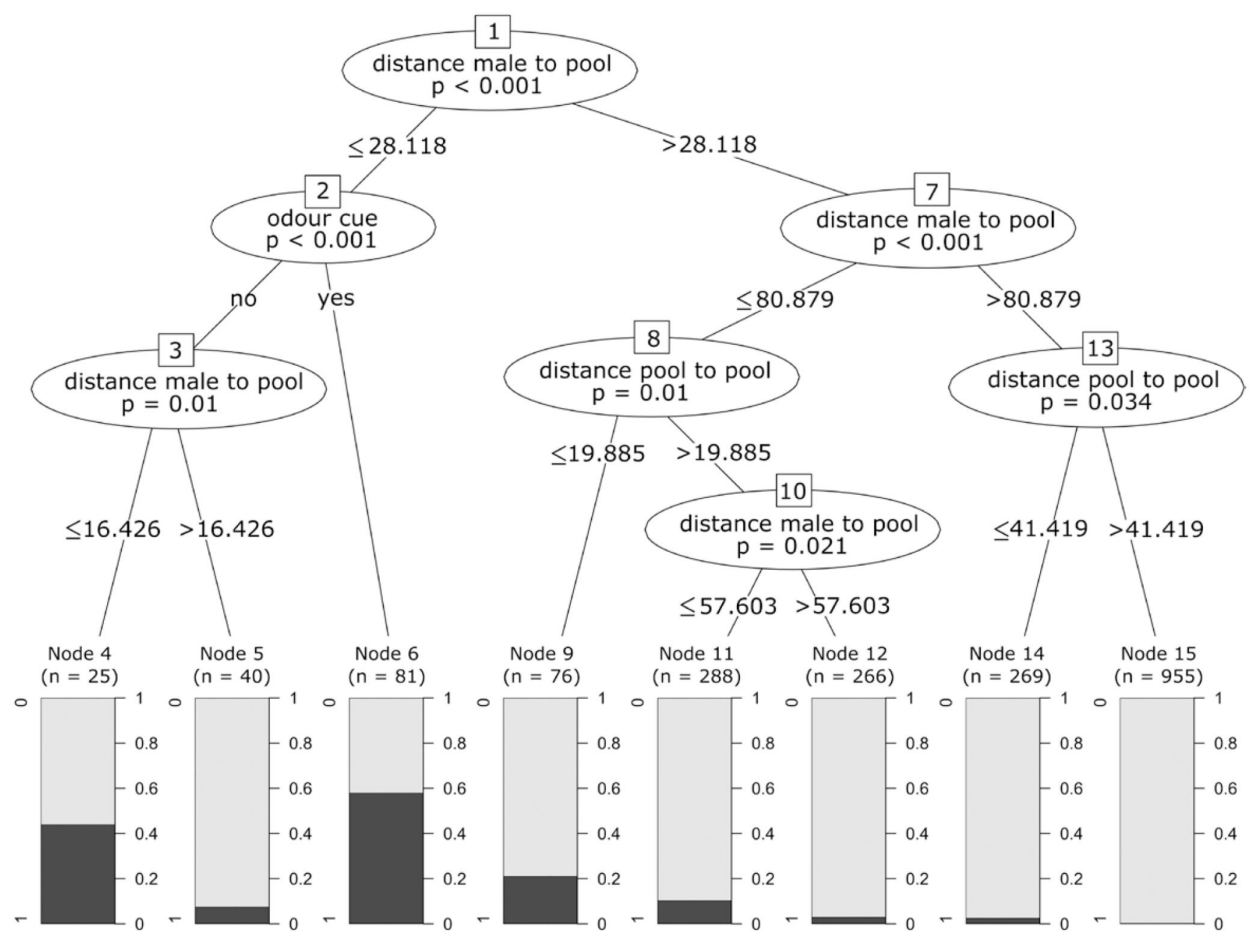
Conditional inference tree examining where male poison frogs choose to deposit their tadpoles. Pools that did receive a deposition (1 in the barplot) versus those that did not (0 in the barplot) were best classified according to 3 categories. The highest deposition frequency (58%) was observed for pools that were in close spatial proximity to a male’s territory and that had odor cues (node 6). “distance male to pool” represents the distance between the new pool used for deposition and the male’s territory. “odour cue” represents the treatment of the pool (with or without odor cue). “distance pool to pool” represents the distance between the new pool and the closest old pool (used or not by the frog before).

The likelihood ratio test revealed that the full model explained the variation in the likelihood to deposit tadpoles better than the null model (v2 = 193.09, *df* = 4, *P* < 0.01). The full model showed that all 3 fixed effects significantly affected the probability of deposition in a given pool. The deposition likelihood of a clutch significantly increased with decreasing distance of a new pool to the father’s territory as well as to the closest old pool, and with the presence of odor cues (Figure 2B, C, Supplementary Table 3). The conditional inference tree analysis confirmed the relative importance ranking of the fixed effects and showed that the distance of a new pool to the father’s territory (*P* < 0.001; Figure 3), followed by the treatment (*P* < 0.001; Figure 3), and the distance between the pool and the closest old pool (*P =* 0.01; Figure 3) were significant predictors for deposition probability. Pools that were close to the male’s territory (closer than 28 m; Figure 3) and that had odor cues were most likely to receive tadpoles (58.0% of the pools that fell into this category were used for deposition; Figure 3, node 6). In contrast, pools that were far from a male’s territory (farther than 81 m; Figure 3) and far from an old pool (farther than 41 m; Figure 3) were least likely to receive tadpoles (Figure 3, node 15).

### Influence of the interplay between personality traits and external cues on paternal care

In both full and splitted models, we found no influence of the interaction between personality traits and treatment or distance between the pool to the male’s territory on pool use. Precisely, the number of new pools used or the likelihood of discovering a new pool within 45 days were not affected by an interaction between exploration or boldness and either treatment or distance between the pool and the male’s territory (Supplementary Tables 4 and 5).

## Discussion

In the present study, we used an entire free-ranging population of the neotropical brilliant-thighed poison frog *A. femoralis* to investigate the role of the interplay between external cues (specifically olfactory cues) and personality traits (specifically exploration and boldness) on the ability of individual males to discover and use novel reproductive resources.

While there is empirical evidence for the interplay between personality and reproductive success in several taxa (for a review see Smith and Blumstein 2008), there is still limited knowledge on the mechanisms of how personality traits translate into fitness (Dingemanse and Réale 2013). One hypothesis is that personality traits influence parental care which in turn can influence fitness (Mutzel et al. 2013). For example, males of the common goby *Pomatoschistus microps* with a more active personality type are less efficient at parental care because they cannibalize more eggs, thereby reducing their reproductive success, compared to less active males (Vallon et al. 2016). We expected *A. femoralis* males with higher levels of exploration and boldness to increase their reproductive output by using a higher number of rearing sites or being more efficient at discovering new rearing sites. Indeed, males’ personality seems to influence reproductive output (i.e., the number of their tadpoles who survive until adulthood; Peignier et al. unpublished data) in *A. femoralis*. However, we found no correlation between levels of exploration or boldness and pool use at the between-individual levels.

The field of animal personality has received a lot of criticism, mainly because there is no consensus about whether behaviors measured in artificial setups (such as the NET) could reflect reliable natural behaviors of the species (cf. Beekman and Jordan 2017). The absence of correlation between exploration or boldness levels and pool use could suggest that the behaviors that we measured in the NET do not represent natural explorative or boldness-related behaviors of *A. femoralis* males. However, a previous study performed in a lab population of *A. femoralis* showed that exploration behaviors measured in the NET indeed reflect natural exploration-related behaviors expressed in the context of territory settlement (Bégué et al. 2023). In this species, exploration is probably more important in early life, when subadult individuals need to explore their environment to find a territory to settle and discover resources, while pool use later on in life is seemingly less affected by exploration levels and probably more motivated by immediate cues.

Previous findings have shown that male *A. femoralis* use spatial memory to navigate in their environment (Pašukonis et al. 2013, 2014, 2016). Males rely on experience and are able to quickly find their way back to their territory when translocated (Pašukonis et al. 2013, 2014). Moreover, males also know the location of several different deposition sites in the area (Pašukonis et al. 2016). These findings suggest that males have an accurate representation of their familiar surroundings. It is, therefore, possible that exploration and boldness do not matter for tadpole deposition in a familiar environment. In turn, activity, as a personality trait, is defined as “the general level of activity of an individual” in a non-novel environment (Réale et al. 2007). Removing known deposition sites probably only slightly changed the familiar environment but did not create a sufficiently novel environment for exploration levels to matter in this context. In addition, our results show that individuals tend to deposit more frequently in new pools that are located closer to their territory. The area surrounding their territory is likely an area that individuals know very well, and deposition within this area might be therefore influenced by activity, rather than exploration or boldness. Future studies should investigate the influence of the individual activity level on resource discovery and use in *A. femoralis*.

In the present study we found that males are more likely to find and use pools that are closer to their territory or to old rearing sites, and pools with olfactory cues. The reason for a greater utilization of closer deposition sites can be understood based on fundamental considerations, assuming that *A. femoralis* males exhibit central-place territoriality. In this scenario, higher activity levels closer to the center of the territory lead to the faster discovery of closer pools. Furthermore, previous studies have shown that *A. femoralis* males rely on spatial memory in different contexts, which likely explains the greater use of new pools that are located closer to old rearing sites. Indeed, there is evidence for natal philopatry in males, with males returning to their natal pools for tadpole deposition, even when closer pools are available (Ringler et al. 2018). In addition, males are known to rely on spatial memory to retrieve known deposition sites (Pašukonis et al. 2016). In our study, we observed that males used fewer new rearing sites (1.54 ± 0.71 *SD*, range = 1−4) compared to old rearing sites (2.20 ± 1.56 *SD*, range = 1−7) but rather went back to the same new pools to deposit their clutches. This supports the idea that males make use of spatial memory in the context of tadpole deposition (Pašukonis et al. 2016; Ringler et al. 2018; Serrano-Rojas and Pašukonis 2021). Finally, the importance of stagnant water and/or tadpole odor cues for finding new rearing sites in *A. femoralis* males has already been identified in previous studies (Pašukonis et al. 2016; Serrano-Rojas and Pašukonis 2021). In our study, we confirmed these findings, but the effect of stagnant water and tadpole odor cannot be disentangled, and both cues might have influenced tadpole deposition. Tadpole transport is presumably quite costly for the transporting parent, as it increases risks of predation, and the loss of the territory and/or mating opportunities (Pough and Taigen 1990; Wells 2007; Carvajal-Castro et al. 2021; Pašukonis et al. 2022). Consequently, males should minimize the time and effort associated with tadpole transport (Ringler et al. 2013b; Beck et al. 2017). Our findings, together with previous studies, indicate that male *A. femoralis* have developed a highly efficient strategy, combining spatial learning and memory, and the use of external cues to find new rearing sites when deciding where to deposit their offspring.

We did not find evidence for an interplay between personality traits and external cues in the context of pool use. Overall our results suggest that a combination of availability, odor cues, and the new pools being close to a male’s territory or to known rearing sites affects which and how new sites are used for tadpole deposition. While the repeated use of known sites has been demonstrated in previous studies (Erich et al. 2015; Pašukonis et al. 2016; Ringler et al. 2018), and new deposition sites come with new unknowns (e.g., desication risk, predator presence, etc.) the more common condition for *A. femoralis* males is the use of ephemeral new sites. Therefore, we assume that our experimental setup ressembled the one that frogs encounter at the beginning of the breeding season, when no pools have been used yet. The ability to discover and use new resources quickly is primordial in highly dynamic environments, such as the tropical rainforests, particularly at the spatial scale of our study species. Thus, individuals should rely on strong and reliable cues to make their decisions. We performed our study in the peak of the rearing season, when most males had clutches that needed to be transported. With the sudden removal of all rearing sites, males were under high pressure to find new rearing sites quickly. In this context, odor cues were probably the most reliable clues to find new deposition sites and prevent total clutch loss.

Between-individual differences in decision-making can be explained by consistent between-individual differences in behavior (Carter et al. 2013), and/or by differences in the use of available external cues (Budaev et al. 2019). However, we still have limited information in which contexts external cues are more important and thus override any potential effect of animal personality. In our study, odor cues associated with deposition sites likely overrode any effect of behavioral traits when discovering and using new resources. Future studies should focus on understanding in which contexts external cues and behavioral traits act together to influence decision-making, and in which contexts one factor overrides the other.

## Supplementary Material

Supplementary Materials

## Data Availability

The datasets generated during and/or analyzed during the current study are available in the Open Science Framework repository: https://osf.io/pk2mf/?view_only=d1b58c1e16ca4b54900d0a0ff52a2e89
