# Supplementary material for: Odor cues rather than personality affect tadpole deposition in a neotropical poison frog
Source: Curr Zool. Author manuscript; Available in PMC 2024 Jul 18. (PMC7616257; doi:10.1093/cz/zoad042)
Supplement: Supplementary Materials [file EMS196912-supplement-Supplementary_Materials.docx]

**Odour cues rather than personality affect tadpole deposition in a Neotropical poison frog**

Mélissa Peignier^a,b,*^, Max Ringler^a,c,d,e^, & Eva Ringler^a,b^

^a^ Division of Behavioural Ecology, Institute of Ecology and Evolution, University of Bern, Switzerland

^b^ Messerli Research Institute, University of Veterinary Medicine Vienna, Austria

^c^ Department of Behavioral and Cognitive Biology, University of Vienna, Austria

^d^ Department of Evolutionary Biology, University of Vienna, Austria

^e^ Institute of Electronic Music and Acoustics, University of Music and Performing Arts Graz, Austria

**Prior used in the bivariate models**

prior1 🡨 list(R = list(V = diag(c(0.0001, 0.0001),2,2), nu = 0.002, fix = 1),

G = list(G1 = list(V = diag(2), nu = 2,

alpha.mu = rep(0,2),

alpha.V = diag(25^2,2,2))))

**Supplementary Table 1.** Variance Inflation Factors (VIF) of the generalized linear mixed effect models investigating the role of the interplay between external cues and personality traits on parental care. Values above 5 indicate multicollinearity.

|  | **exploration score** | **boldness score** | **treatment** | **distance between pool and male’s territory** | **exploration* treatment** | **exploration* distance between pool and male’s territory** | **boldness* treatment** | **boldness* distance between pool and male’s territory** |
| --- | --- | --- | --- | --- | --- | --- | --- | --- |
| number of new pools used | 6.65 | 6.08 | 1.16 |  | 6.08 |  | 5.55 |  |
| number of new pools used | 1.63 | 1.69 |  | 1.17 |  | 1.51 |  | 1.78 |
| discovered within 45 days (1/0) | 14.92 | 8.98 | 1.26 |  | 13.68 |  | 7.91 |  |
| discovered within 45 days (1/0) | 1.29 | 1.28 |  | 1.18 |  | 1.53 |  | 1.56 |

**Supplementary Table 2.** Between-individual covariance and slope between the factor score extracted from structural equation models for the personality traits exploration and boldness and measurements of parental performance. We present the mean and 95% credible intervals.

|  |  | **between-individual covariance** | **between-individual slope** |
| --- | --- | --- | --- |
| **behaviour** | **measures of parental performance** | **mean (95% Credible Interval)** | |
| exploration score | number of new pools used | -0.04 (-0.20; 0.13) | -0.20 (-0.99; 0.64) |
|  | number of old pools used | -0.25 (-0.78; 0.19) | -0.13 (-0.35; 0.10) |
|  | discovered within 45 days (1/0) | -0.38 (-1.19; 0.17) | -0.10 (-2.60; 5.62) |
|  | average distance to new pools used | -0.13 (-0.40; 0.10) | -0.28 (-0.76; 0.20) |
|  | average distance to old pools used | -0.08 (-0.34; 0.13) | -0.22 (-0.82; 0.36) |
| boldness score | number of new pools used | -0.04 (-0.15; 0.07) | -0.20 (-74; 0.34) |
|  | number of old pools used | 0.19 (-0.14; 0.55) | -0.10 (-0.07; 0.27) |
|  | discovered within 45 days (1/0) | -0.14 (-0.58; 0.50) | -0.34 (-1.14; 0.29) |
|  | average distance to new pools used | -0.08 (-0.28; 0.09) | -0.17 (-0.51; 0.18) |
|  | average distance to old pools used | -0.03 (-0.24; 0.17) | -0.08 (-0.57; 0.45) |

**Supplementary Table 3.** Results of the generalized linear mixed effect models investigating the influence of external cues on tadpole deposition likelihood. We present estimates, standard-error (SE), standard-deviation (SD), p-values and 95% confidence intervals (95%CI). Significant results (p-value < 0.05) are written in bold. N = 2000 observation on 41 males and 90 clutches.

|  | **Deposition likelihood** | | | |
| --- | --- | --- | --- | --- |
| **Fixed effects** | *Estimate* | *SE* | *p-value* | *95%CI* |
| (intercept) | 0.08 | 0.33 | 0.813 | -0.57, 0.72 |
| treatment | 1.11 | 0.22 | **< 0.001** | 0.68, 1.56 |
| distance between pool and male’s territory | -0.05 | 0.00 | **< 0.001** | -0.05, -0.04 |
| distance between pool and closest old pool | -0.01 | 0.01 | **0.026** | -0.03, -0.002 |
| **Random effects** | *Estimate* | *SD* |  |  |
| clutch ID:male ID | 0 | 0 |  |  |
| maleID | 0 | 0 |  |  |

**Supplementary Table 4.** Results of the full generalized linear mixed effect models investigating the role of the interplay between external cues and personality traits on parental care. Exploration and boldness are factor scores extracted from structural equation models. We present estimates, standard-error (SE), standard-deviation (SD), and p-values. The fixed effect ‘dist.male-pool’ represents the distance between the pool and the male’s territory. Significant results (p-value < 0.05) are written in bold. N = 122 depositions from 40 males.

|  | **Number of new pools used** | | | **Number of new pools used** | | | **Likelihood to discover a pool within 45 days** | | | **Likelihood to discover a pool within 45 days** | | |
| --- | --- | --- | --- | --- | --- | --- | --- | --- | --- | --- | --- | --- |
| **Fixed effects** | *Estimate* | *SE* | *p-value* | *Estimate* | *SE* | *p-value* | *Estimate* | *SE* | *p-value* | *Estimate* | *SE* | *p-value* |
| (intercept) | 0.53 | 0.16 | **0.001** | 0.51 | 0.09 | **< 0.001** | -12.82 | 4.21 | **0.002** | -12.68 | 3.15 | **< 0.001** |
| exploration score | -0.05 | 0.21 | 0.814 | 0.06 | 0.1 | 0.544 | -2.19 | 8.75 | 0.802 | -0.99 | 2.39 | 0.678 |
| treatment | -0.02 | 0.18 | 0.916 |  |  |  | -0.07 | 4.2 | 0.987 |  |  |  |
| boldness score | -0.07 | 0.28 | 0.804 | -0.13 | 0.15 | 0.372 | 1.4 | 7.66 | 0.855 | 0.69 | 2.91 | 0.812 |
| exploration score * treatment | 0.14 | 0.24 | 0.565 |  |  |  | 1.65 | 8.68 | 0.849 |  |  |  |
| boldness score * treatment | -0.05 | 0.32 | 0.883 |  |  |  | -1.02 | 8.08 | 0.900 |  |  |  |
| dist.male-pool |  |  |  | 0.02 | 0.08 | 0.806 |  |  |  | -0.33 | 2.45 | 0.893 |
| exploration score * dist.male-pool |  |  |  | -0.01 | 0.11 | 0.917 |  |  |  | 1.31 | 2.94 | 0.656 |
| boldness score * dist.male-pool |  |  |  | 0.06 | 0.16 | 0.692 |  |  |  | -0.94 | 3.96 | 0.813 |
| **Random effects** | *Estimate* | *SD* |  | *Estimate* | *SD* |  | *Estimate* | *SD* |  | *Estimate* | *SD* |  |
| maleID | 0.06 | 0.24 |  | 0.06 | 0.25 |  | 3549 | 59.57 |  | 3261 |  | 57.51 |

**Supplementary Table 5.** Results of the generalized linear mixed effect models that we splitted based on the VIF values. These models investigates the role of the interplay between external cues and personality traits on parental care. Exploration and boldness are factor scores extracted from structural equation models. We present estimates, standard-error (SE), standard-deviation (SD), and p-values. Significant results (p-value < 0.05) are written in bold. N = 122 depositions from 40 males.

|  | **Number of new pools used** | | | **Number of new pools used** | | | **Likelihood to discover a pool within 45 days** | | | **Likelihood to discover a pool within 45 days** | | |
| --- | --- | --- | --- | --- | --- | --- | --- | --- | --- | --- | --- | --- |
| **Fixed effects** | *Estimate* | *SE* | *p-value* | *Estimate* | *SE* | *p-value* | *Estimate* | *SE* | *p-value* | *Estimate* | *SE* | *p-value* |
| (intercept) | 0.52 | 0.16 | **0.001** | 0.51 | 0.16 | **0.001** | -12.91 | 4.05 | **0.001** | -13.18 | 4.24 | **0.002** |
| exploration score | -0.09 | 0.15 | 0.555 |  |  |  | -0.86 | 3.88 | 0.825 |  |  |  |
| treatment | -0.03 | 0.18 | 0.866 | 0.00 | 0.18 | 0.988 | 0.09 | 3.78 | 0.981 | 0.21 | 4.15 | 0.960 |
| boldness score |  |  |  | -0.12 | 0.20 | 0.549 |  |  | 0.911 | -0.24 | 4.12 | 0.953 |
| exploration score * treatment | 0.13 | 0.18 | 0.460 |  |  |  | 0.48 | 4.24 |  |  |  |  |
| boldness score * treatment |  |  |  | 0.06 | 0.23 | 0.791 |  |  |  | 0.30 | 4.94 | 0.951 |
| **Random effects** | *Estimate* | *SD* |  | *Estimate* | *SD* |  | *Estimate* | *SD* |  | *Estimate* | *SD* |  |
| maleID | 0.06 | 0.25 |  | 0.07 | 0.26 |  | 3624 | 60.2 |  | 3725 | 61.04 |  |
